# Disease activity and low physical activity associate with number of hospital admissions and length of hospitalisation in patients with rheumatoid arthritis

**DOI:** 10.1186/ar3390

**Published:** 2011-06-29

**Authors:** George S Metsios, Antonios Stavropoulos-Kalinoglou, Gareth J Treharne, Alan M Nevill, Aamer Sandoo, Vasileios F Panoulas, Tracey E Toms, Yiannis Koutedakis, George D Kitas

**Affiliations:** 1 Department of Physical Activity, Exercise and Health, University of Wolverhampton, Gorway Road, Walsall, WS13BD, West Midlands, UK; 2 Department of Rheumatology, Dudley Group of Hospitals NHS Trust, Russell's Hall Hospital, Pensnett Road, DY12HQ, Dudley, West Midlands, UK; 3 Research Institute in Physical Performance and Rehabilitation, Centre for Research and Technology - Thessaly, Trikala, Karies, GR42100, Greece; 4 Department of Psychology, University of Otago, St David Street, Dunedin North 9016, New Zealand; 5 ARC Epidemiology Unit, Manchester Metropolitan University, Oxford Road, M156BH, Manchester, UK

## Abstract

**Introduction:**

Substantial effort has been devoted for devising effective and safe interventions to reduce preventable hospital admissions in chronic disease patients. In rheumatoid arthritis (RA), identifying risk factors for admission has important health policy implications, but knowledge of which factors cause or prevent hospital admissions is currently lacking. We hypothesised that disease activity/severity and physical activity are major predictors for the need of hospitalisation in patients with RA.

**Methods:**

A total of 244 RA patients were assessed for: physical activity (International Physical Activity Questionnaire), RA activity (C-reactive protein: CRP; disease activity score: DAS28) and disability (Health Assessment Questionnaire: HAQ). The number of hospital admissions and length of hospitalisation within a year from baseline assessment were collected prospectively.

**Results:**

Disease activity and disability as well as levels of overall and vigorous physical activity levels correlated significantly with both the number of admissions and length of hospitalisation (*P *< 0.05); regression analyses revealed that only disease activity (DAS28) and physical activity were significant independent predictors of numbers of hospital admissions (DAS28: (exp(B) = 1.795, *P *= 0.002 and physical activity: (exp(B) = 0.999, *P *= 0.046)) and length of hospitalisation (DAS28: (exp(B) = 1.795, *P *= 0.002 and physical activity: (exp(B) = 0.999, *P *= 0.046). Sub-analysis of the data demonstrated that only 19% (*n *= 49) of patients engaged in recommended levels of physical activity.

**Conclusions:**

This study provides evidence that physical activity along with disease activity are important predictors of the number of hospital admissions and length of hospitalisation in RA. The combination of lifestyle changes, particularly increased physical activity along with effective pharmacological therapy may improve multiple health outcomes as well as cost of care for RA patients.

## Introduction

Rheumatoid arthritis (RA), the most common chronic inflammatory arthritis, typically leads to physical disability and worse quality of life. Its associated health effects result in significant treatment costs compared to patients with other chronic diseases or the general population [1,2], including hospitalisation costs due to the increased number of admissions, which create a large economic burden [1]. The introduction of biological treatments for RA has increased drug-related costs [3,4], but reduced the need for hospital admissions [5]. However, there may be several other contributors to hospital admissions in patients with RA.

Investigating the ways that non-pharmacological interventions may improve RA outcomes, most importantly increased physical activity, has been an interesting challenge for rheumatology health professionals. This is because it is difficult to overcome the pain and physical disability barriers that accompany this disease and convince patients that exercise and/or increased physical activity will improve disease outcomes [6]. Because of this, it is not surprising that there is a high prevalence of physical inactivity in regular clinical RA patients in 21 countries [7]. Nevertheless, pooled evidence reveals that participation in exercise has beneficial effects on RA disease activity and severity as it inhibits disease progression without inducing flares [8]. Moreover, increased physical activity may improve the cost-effectiveness of treatment particularly in patients with increased cardiovascular risk [9], as is the case for RA patients [10].

The number of hospital admissions and length of hospitalisation represent very important parameters that may affect cost of treatment and quality of life. Therefore, substantial effort has being devoted to devise effective and safe interventions to reduce preventable hospital admissions in patients with many diseases [9]. In RA, identifying risk factors for admission has important health policy implications, but knowledge of which factors associate with hospital admissions and/or length of hospitalisation is currently lacking. Such knowledge is crucial given that, identifying predictors of hospital admissions may help focus provision of care on the individuals at risk and allow targeted interventions. The main aim of this study was to investigate whether RA disease activity and disability and/or involvement in physical activity are significant predictors of the number of hospital admissions and length of hospitalisation in RA patients.

## Materials and methods

### Participants

Two hundred and forty-four consecutive patients with RA, meeting the revised RA American College of Rheumatology classification criteria [11], were recruited from the clinics of the Dudley Group of Hospitals NHS Foundation Trust, UK. Prior to participation, verbal and written information about the study was given to the participants. Upon deciding to participate, a written informed consent was signed and a follow-up visit was arranged at the Rheumatology Clinical Research Unit. The study was approved by the Black Country research ethics committee and research and development directorate.

### Procedures

Patients visited our laboratory following a 12 h overnight fast. On that day, we have initially collected our patients' demographic data followed by evaluation of anthropometric characteristics. Height was assessed via a Seca Stadiometer 208, whereas weight, body mass index, body fat and fat-free mass were measured via bioelectrical impedance (Tanita BC 418 MA, Tanita Corporation, Tokyo, Japan). Using standardised laboratory procedures, contemporary serological inflammatory load and clinical disease activity were assessed by the erythrocyte sedimentation rate (ESR), C-reactive protein (CRP) and the Disease Activity Score-28 (DAS28). Functional disability was self-reported via the Health Assessment Questionnaire (HAQ). Disease duration was recorded from reviews of the participants' hospital notes.

The long version of the international physical activity questionnaire (IPAQ) was used to assess levels of the patients' physical activity. The IPAQ is suitable for patient populations [12] as it is divided in specific parts, each addressing the physical activities that patients with chronic disease are most likely to perform: job-related, transportation, housework, leisure time, and time spent sitting. Further, the IPAQ utilises as its unit "MET-min/week", where MET is the metabolic and/or energy cost of physical activities.

Data for numbers and reasons for hospital admissions as well as the length of hospitalisation (that is, total days that a patient stayed as an inpatient at the hospital as a result of the admission) per patient were provided by the hospital's information department. Reasons for hospital admission were classified in major categories, including: treatment for RA flare (including treatment for severe pain and joint aspiration and injection), single or multiple diagnostic tests requiring admission, emergency admissions for other reasons (for example, infections, cardiovascular emergencies), emergency or elective operations (for example, for fractures or joint replacements). Routine visits were not included in the present analyses in order to focus the investigation on care required in addition to routine outpatient monitoring appointments or visits for routine day-case therapy.

### Statistical analyses

Kolmogorov-Smirnov normality tests were utilised to investigate the normal distribution of data. Paired-samples t-test or Mann-Whitney U tests were utilised for comparisons between groups (depending on the normality of the distributions). For correlation coefficient and regression analyses, the number of hospital admissions was dichotomised in "zero to one" and "above one" whereas the length of hospitalisation was dichotomised into "zero" and "one and above"; this approach was adopted due to the severe skew of both these variables. Following dichotomisation, Spearman's rank correlation was used to evaluate the relationships of both these variables with HAQ, DAS28, ESR, CRP, disease duration, age, overall and vigorous physical activity. The number of hospital admissions and length of hospitalisation (again both as dichotomous variables), were used as dependent variables in binary logistic regression analyses to assess the effect of various different predictor variables (HAQ, DAS28, ESR, CRP, disease duration, age, overall and vigorous physical activity). Further bivariate analyses and regressions were run to examine predictors of the demographic or RA-related variables associated with hospital admissions and length of hospitalisation following Baron and Kenny's criteria of mediation effects [13]. All statistical analyses were conducted using SPSS (version 16, Chicago, IL, USA).

## Results

The general characteristics of the patients appear in Table 1. The level of physical activity (PA) of this patient group was 1,550 (989.5 to 2,175.0) MET-minutes/week. From our total sample size (*n *= 244), 39% (*n *= 94) were admitted to the hospital within one year. The number and frequencies for hospital admissions appear in Table 2. Regarding the length of hospitalisation, 32 patients (out of the 94 patients admitted) stayed as inpatients at the hospital after they were admitted. The length of hospitalisation for these 32 patients was: a) between one to five days for eight patients (25% of the 32 patients), b) between six and 10 days for six patients (19% of the 32 patients), and c) above 10 days for 18 patients (56% of the 32 patients), whereas the reasons for hospitalisation were: infusions/injections (*n *= 20), pain (*n *= 2), joint replacement (*n *= 4), fractures (*n *= 2), respiratory (*n *= 2) and cardiovascular (*n *= 2) complications.

**Table 1:** Demographic, anthropometric and clinical characteristics of the study population number

*General demographics*			
	**Males (*n *= 70)**	**Females (*n *= 174)**	**Total**
Physical activity (MET-minutes/week)	1,674.5 (982.5 to 2,479.2)	1,470.0 (993.0 to 2,082.5)	1,550 (989.5 to 2,175.0)
Age (years)	62.1 (55.8 to 68.7)	62.3 (52.8 to 70.2)	62.1 (53.8 to 69.4)
Smoking status			
current smokers n (%)	14 (20.9%)	31 (18.1%)	45 (18.9%)
***Anthropometric ***			
Height (cm)	172.9 ± 7.1	160.7 ± 6.9*	164.2 ± 8.8
Weight (kg)	81.7 (73.1 to 93.0)	70.8 (61.6 to 81.3)**	73.8 (64.9 to 84.0)
Body Mass Index (kg/m^2^)	27.1 (25.0 to 30.3)	26.7 (24.1 to 31.7)	27.0 (24.4 to 30.8)
Fat-free mass (kg)	58.6 (53.1 to 65.4)	43.4 (39.1 to 46.9)**	45.9 (41.4 to 53.1)
Fat mass (%)	28.6 (22.2 to 31.8	38.8 (34.6 to 43.1)**	36.2 (29.7 to 40.8)
***RA characteristics***			
***General characteristics***			
Rheumatoid factor positive n (%)	45 (72.6%)	118 (77.1%)	163 (75.8%)
Disease duration (years)	9.0 (3.5 to 18.0)	11.0 (4.0 to 20.0)	11.0 (4.0 to 19.0)
***Disease activity***			
C-Reactive protein (mg/L)	11.5 (6.0 to 22.2)	8.0 (5.0 to 20.0)	9.0 (5.0 to 21.0)
ESR (mm/1 hr)	23.0 (5.0 to 39.0)	22.0 (12.2 to 39.0)	23.0 (10.0 to 39.0)
Disease activity score 28	4.3 ± 1.4	4.2 ± 1.5	4.2 ± 1.4
***Disability***			
Health assessment Questionnaire	1.2 (0.5 to 2.1)	1.5 (0.5 to 2.1)	1.5 (0.5 to 2.1)
***Medication***			
DMARDs n (%)	65 (92.9%)	144 (83.2%)*	209 (86%)
Methotrexate n (%)	41 (58.6%)	95 (54.9%)	136 (56%)
antiTNFα n (%)	6 (8.6%)	24 (13.9%)	30 (12.3%)
leflunomide n (%)	1 (1.4%)	10 (5.8%)	11 (4.5%)
prednisolone n (%)	30 (42.9%)	51 (29.5%)	81 (33.3%)
NSAID n (%)	16 (22.9%)	30 (17.3%)	46 (18.9%)
Cholesterol-lowering n (%)	16 (22.9%)	28 (16.2%)	44 (18.1%)

Results expressed as number (percentages), median (interquartile range) or mean ± SD as appropriate.

ESR, erythrocyte sedimentation rate, DMARDs, disease modifying anti rheumatic drugs, NSAID, non-steroid anti-inflammatory drugs

**Table 2:** Total number of hospital admissions across one year

Number of Hospital Admissions	Number (%) of RA patients
0	150 (61%)
1 to 5	77 (31%)
6 to 10	5 (2%)
11 to 20	4 (2%)
> 20	8 (4%)

### Correlations

#### Number of admissions

Functional disability (HAQ), disease activity (DAS28), inflammatory markers (CRP and ESR) significantly correlated with the number of admissions (HAQ: rho = 0.214, *P *= 0.001; DAS28: rho = 0.183, *P *= 0.008; CRP: rho = 0.169, *P *= 0.008; ESR: rho = 0.161, *P *= 0.012) whereas this was not the case for disease duration or patient's age (*P *< 0.05). Frequency/amount (in MET-minutes/week) of overall physical activity and 'vigorous' physical activity demonstrated significant negative correlations with the number of admissions (rho = -0.262, *P *< 0.001 and rho = -0.270, *P *< 0.001, respectively).

#### Length of hospitalisation

HAQ, DAS28 and inflammatory markers (CRP and ESR) also revealed significant correlations (HAQ: rho = 0.280, *P *< 0.001; DAS28: rho = 0.225, *P *= 0.001; CRP: rho = 0.147, *P *= 0.022; ESR: rho = 0.249, *P *< 0.001). Physical activity and vigorous physical activity were again inversely correlated with length of hospitalisation (rho = -0.231, *P *< 0.001 and rho = -0.295, *P *< 0.001, respectively). No other parameters revealed significant correlations with length of hospitalisation.

### Regression analyses

**Figure 1:**
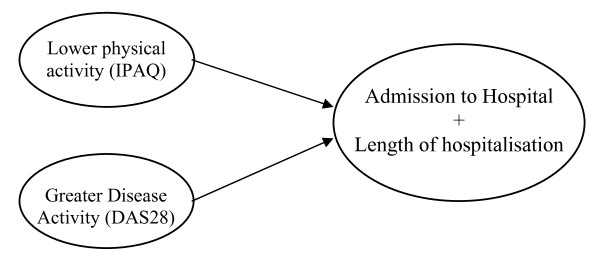
**Variables associated with RA-related hospital admission over the course of a year**.

We have performed two different binary logistic regressions for the numbers of hospital admission as well as the length of hospitalisation, respectively. Based on the results from the correlations, in both models we used as independent variables HAQ, DAS28, CRP and ESR in an initial forward entry step, followed by overall physical activity and 'vigorous' physical activity on a final step. In the first step, only DAS28 was a significant predictor (exp(B) = 1.437, *P *= 0.005) whereas in the final step, both overall physical activity (exp(B) = 0.999, *P *= 0.005) and DAS28 (exp(B) = 1.397, *P *= 0.011) were both significant predictors of the number of hospital admissions (Figure 1). Similarly, DAS28 significantly predicted length of hospitalisation (exp(B) = 1.815, *P *= 0.001) whereas during the final step both DAS28 and overall physical activity were significant predictors ((exp(B) = 1.795, *P *= 0.002 and (exp(B) = 0.999, *P *= 0.046).

In a sub-analysis of our data we found that only 19% (*n *= 49) of participants were engaged in recommended levels of physical activity (≥ 5 times/week for ≥ 30 minutes). This group of participants had a significantly lower number of admissions compared to the remaining patients (physically active: 0.0 (0.0 to 0.0) vs. inactive: 0.0 (0.0 to 2.0), *P *< 0.001). In addition, patients achieving recommended levels of physical activity had significantly less swollen joints (3.0 (1.0 to 6.0) vs. 4.0 (2.0 to 8.0), *P *= 0.02] as well as significantly better physical function (HAQ: 1.0 (0.0 to 2.0) vs. 2.0 (0.0 to 2.0), *P *= 0.001). However, this group was significantly younger (physically active vs. inactive: age 56.8 ± 13.1 vs. 62.9 ± 11.5 years, *P *= 0.001) but did not have significantly different disease duration compared to the physically inactive group (*P *> 0.05). In an additional logistic regression, it was found that both younger age and lower CRP were significant predictors of whether participants met the recommendations for physical activity (exp(B) = 0.55, *P *= 0.02), regardless of their DAS28 and HAQ scores.

## Discussion

This study investigated for the first time the impact of physical activity levels on hospital admissions and length of hospitalisation over one year in patients with RA. Our results revealed that disease activity and physical activity are both significant predictors of these two variables.

Studies with RA patients reveal that, due to the high prevalence of co-morbidities [5], patients feel uncertain about the outcomes of their disease and hence, admission to the hospital may have deleterious effects, particularly in patients with early disease [14]. Hospitalisation may lead to negative self-esteem and loss of privacy [14] and may have a significant, lasting adverse impact on the quality of life of the patient. It also associates with very high costs to the health system. Hence, it is important to identify strategies that may improve overall management and reduce hospitalisation in this patient group.

To this end, improved pharmacological therapy for RA, particularly after the introduction of biological medication with anti-TNFa agents, has significantly improved disease management and appears to reduce hospital admissions and lengths of stay [15], but it also increased direct drug costs [2,4,16]. A very important factor that may considerably affect RA management is lifestyle change with increased involvement in exercise and/or physical activity. The results from the present study may also suggest beneficial effects of physical activity, both to the individual patients and the healthcare system, by a reduction of the number and length of hospitalisation. However, the cross sectional design adopted herein cannot prove definite causality and it is likely that the number of hospital admissions as well as the length of hospitalisation is mediated by many different factors, which have to be investigated in relevant trials. Our suggestion for a potential association of increased physical activity with reduced admission rates lies in robust research evidence which have consistently shown that regular exercise and physical activity significantly improve RA patient outcomes (by promoting beneficial body composition changes and reducing fatigue), inhibit progression of the disease (by reducing inflammation and increasing muscle mass and bone mineral density), and lead to significantly better cardiovascular health and reduced risk to develop cardiovascular disease [8,17-19].

Previous studies have shown that disease activity significantly influences direct and indirect RA costs [1]. We found that disease activity and disability may also impact upon future admission rates. In fact, we have previously demonstrated that effective treatment enables patients to engage with more active lifestyles and better diet [20]. The combination of increased physical activity and effective medication, therefore, may not only inhibit disease progression thereby improving quality of life, but it may also reduce costs by reducing the need for surgery, and admission to acute and extended care hospitalisation, as well as social service utilization.

The observed physical activity levels herein are significantly lower compared to patients with other chronic diseases, including obesity [21], cancer [22] and osteoarthritis [23]. More importantly, only a fifth (19%) of the total wide-range (in terms of age and disease duration) RA population studied, achieved the recommended levels of physical activity, a significantly reduced number compared to the normal population (approximately 35%) [24]. More importantly, this 19% corresponds mainly to the younger RA patients. Although it is well-established that aerobic capacity is significantly compromised in the RA population [8], our data also demonstrate that RA patients do not achieve the physical activity levels required to minimise their risk for developing cardiovascular disease, inhibit age-related muscle loss, improve quality of life and well-being. Improvement in these parameters is crucial as the prevalence of cardiovascular disease and cachexia is higher in RA than in the normal population [10], partly due to the presence of traditional risk factors [25-28] but also due to the metabolic and vascular effects of persistent high-grade inflammation [29,30]. Moreover, physical ability may be worse due to disease-related processes, although it may be partly improved by effective treatment strategies [20]. Participation in structured exercise programmes is necessary to reversing these phenomena, but this requires patients to be in a controlled environment. Involvement in increased physical activity such as leisure walking, however, is different and requires a different level of determination and commitment given the lack of immediate advice that is available in structured exercise programmes by the instructors. Thus, improving determination to keep active should be a future focus of intervention strategies in order to improve health and quality of life in this population.

One of the important limitations of the present study is the adopted cross-sectional design which is not sufficient to prove a cause-and-effect relationship between the parameters studied. As such, it cannot be ensured that physical activity may have a profound effect on RA, which in turn will result in reduced admission rates or if, in contrast, patients who exercise more have lower disease activity and severity and, hence, they are not admitted to the hospital frequently. Ensuring quality primary care has been recognised as a crucial component in keeping patients with chronic disease out of hospital [31]. It has also been suggested that patients from disadvantaged areas have a higher and prolonged rate of admission [31]. We were not able to standardise for these factors in the present study; all patients came from a relatively distinct geographical area of the UK, which, however, contains a diverse socioeconomic strata and variable access and quality of primary care services. We also did not assess directly either the effects of hospitalisation to quality of life, or the costs incurred as a result of it. On the other hand, the originality of the question, use of validated measures in a consistent fashion, as well as possible mediation or moderation effects, represent important strengths of the study. Clearly, several of the associations found here need to be confirmed in future prospective studies, designed specifically for the purpose.

## Conclusions

This study suggests that disease activity and physical activity are important predictors of the number of hospital admissions as well as length of hospitalisation in RA patients. The combination of lifestyle approaches, in particular increased physical activity, along with effective pharmacological management, is likely to provide superior personal health and health economic outcomes in this population. However, these remain to be investigated in appropriately designed studies.

## Abbreviations

CRP: C-reactive protein; DAS28: Disease Activity Score-28; ESR: erythrocyte sedimentation rate; HAQ: Health assessment questionnaire; IPAQ: International Physical Activity Questionnaire; RA: rheumatoid arthritis.

## Competing interests

The authors declare that they have no competing interests.

## Authors' contributions

GSM, ASK, AS, VFP, YK and TET have contributed substantially in the processes of study design, data acquisition, analyses and interpretation of data. GJT and AMN have contributed in the statistical analyses of the data. GDK has been involved in revising the manuscript critically for its important intellectual concept and also gave the final approval for its publication. All authors read and approved the final manuscript.

## References

[B1] Rat AC Boissier MC Rheumatoid arthritis: direct and indirect costs Joint Bone Spine 2004 71 518 524 10.1016/j.jbspin.2004.01.003 15589432

[B2] Verstappen SM Jacobs JW van der Heijde DM van der Linden S Verhoef CM Bijlsma JW Boonen A Utility and direct costs: ankylosing spondylitis compared with rheumatoid arthritis Ann Rheum Dis 2007 66 727 731 10.1136/ard.2006.061283 17172249 PMC1954669

[B3] Verstappen SM Jacobs JW Kruize AA Ehrlich JC van Albada-Kuipers GA Verkleij H Buskens E Bijlsma JW Trends in economic consequences of rheumatoid arthritis over two subsequent years Rheumatology (Oxford) 2007 46 968 974 10.1093/rheumatology/kem018 17337750

[B4] Witney AG Treharne GJ Tavakoli M Lyons AC Vincent K Scott DL Kitas GD The relationship of medical, demographic and psychosocial factors to direct and indirect health utility instruments in rheumatoid arthritis Rheumatology (Oxford) 2006 45 975 981 10.1093/rheumatology/kel027 16461437

[B5] Treharne GJ Douglas KM Iwaszko J Panoulas VF Hale ED Mitton DL Piper H Erb N Kitas GD Polypharmacy among people with rheumatoid arthritis: the role of age, disease duration and comorbidity Musculoskeletal Care 2007 5 175 190 10.1002/msc.112 17623274

[B6] Munneke M de Jong Z Zwinderman AH Ronday HK van den Ende CH Vliet Vlieland TP Hazes JM High intensity exercise or conventional exercise for patients with rheumatoid arthritis? Outcome expectations of patients, rheumatologists, and physiotherapists Ann Rheum Dis 2004 63 804 808 10.1136/ard.2003.011189 15194575 PMC1755060

[B7] Sokka T Hakkinen A Kautiainen H Maillefert JF Toloza S Mork Hansen T Calvo-Alen J Oding R Liveborn M Huisman M Alten R Pohl C Cutolo M Immonen K Woolf A Murphy E Sheehy C Quirke E Celik S Yazici Y Tlustochowicz W Kapolka D Skakic V Rojkovich B Müller R Stropuviene S Andersone D Drosos AA Lazovskis J Pincus T Physical inactivity in patients with rheumatoid arthritis: data from twenty-one countries in a cross-sectional, international study Arthritis Rheum 2008 59 42 50 10.1002/art.23255 18163412

[B8] Metsios GS Stavropoulos-Kalinoglou A Veldhuijzen van Zanten JJ Treharne GJ Panoulas VF Douglas KM Koutedakis Y Kitas GD Rheumatoid arthritis, cardiovascular disease and physical exercise: a systematic review Rheumatology (Oxford) 2008 47 239 248 18045810 10.1093/rheumatology/kem260

[B9] Roine E Roine RP Rasanen P Vuori I Sintonen H Saarto T Cost-effectiveness of interventions based on physical exercise in the treatment of various diseases: a systematic literature review Int J Technol Assess Health Care 2009 25 427 454 10.1017/S0266462309990353 19845974

[B10] Kitas GD Erb N Tackling ischaemic heart disease in rheumatoid arthritis Rheumatology (Oxford) 2003 42 607 613 10.1093/rheumatology/keg175 12709534

[B11] Arnett FC Edworthy SM Bloch DA McShane DJ Fries JF Cooper NS Healey LA Kaplan SR Liang MH Luthra HS The American Rheumatism Association 1987 revised criteria for the classification of rheumatoid arthritis Arthritis Rheum 1988 31 315 324 10.1002/art.1780310302 3358796

[B12] Craig CL Marshall AL Sjostrom M Bauman AE Booth ML Ainsworth BE Pratt M Ekelund U Yngve A Sallis JF Oja P International physical activity questionnaire: 12-country reliability and validity Med Sci Sports Exerc 2003 35 1381 1395 10.1249/01.MSS.0000078924.61453.FB 12900694

[B13] Baron RM Kenny DA The moderator-mediator variable distinction in social psychological research: Conceptual, strategic and statistical considerations J Pers Soc Psychol 1986 51 1173 1182 3806354 10.1037//0022-3514.51.6.1173

[B14] Edwards J Mulherin D Ryan S Jester R The experience of patients with rheumatoid arthritis admitted to hospital Arthritis Rheum 2001 45 1 7 10.1002/1529-0131(200102)45:1<1::AID-ANR77>3.0.CO;2-Q 11308053

[B15] Wilson AS Kitas GD Carruthers DM Reay C Skan J Harris S Treharne GJ Young SP Bacon PA Computerized information-gathering in specialist rheumatology clinics: an initial evaluation of an electronic version of the Short Form 36 Rheumatology (Oxford) 2002 41 268 273 10.1093/rheumatology/41.3.268 11934962

[B16] Collings S Highton J Changing patterns of hospital admissions for patients with rheumatic diseases N Z Med J 2002 115 131 132 12013305

[B17] Metsios GS Stavropoulos-Kalinoglou A Panoulas VF Wilson M Nevill AM Koutedakis Y Kitas GD Association of physical inactivity with increased cardiovascular risk in patients with rheumatoid arthritis Eur J Cardiovasc Prev Rehabil 2009 16 188 194 10.1097/HJR.0b013e3283271ceb 19238083

[B18] de Jong Z Munneke M Zwinderman AH Kroon HM Ronday KH Lems WF Dijkmans BA Breedveld FC Vliet Vlieland TP Hazes JM Huizinga TW Long term high intensity exercise and damage of small joints in rheumatoid arthritis Ann Rheum Dis 2004 63 1399 1405 10.1136/ard.2003.015826 15479889 PMC1754798

[B19] Metsios GS Stavropoulos-Kalinoglou A Sandoo A van Zanten JJ Toms TE John H Kitas GD Vascular function and inflammation in rheumatoid arthritis: the role of physical activity Open Cardiovasc Med J 4 89 96 20361002 10.2174/1874192401004020089 PMC2847820

[B20] Metsios GS Stavropoulos-Kalinoglou A Douglas KM Koutedakis Y Nevill AM Panoulas VF Kita M Kitas GD Blockade of tumour necrosis factor-alpha in rheumatoid arthritis: effects on components of rheumatoid cachexia Rheumatology (Oxford) 2007 46 1824 1827 10.1093/rheumatology/kem291 18032540

[B21] Tehard B Saris WH Astrup A Martinez JA Taylor MA Barbe P Richterova B Guy-Grand B Sorensen TI Oppert JM Comparison of two physical activity questionnaires in obese subjects: the NUGENOB study Med Sci Sports Exerc 2005 37 1535 1541 10.1249/01.mss.0000177464.68521.3b 16177606

[B22] Johnson-Kozlow M Sallis JF Gilpin EA Rock CL Pierce JP Comparative validation of the IPAQ and the 7-Day PAR among women diagnosed with breast cancer Int J Behav Nutr Phys Act 2006 3 7 10.1186/1479-5868-3-7 16579852 PMC1468425

[B23] Rosemann T Kuehlein T Laux G Szecsenyi J Factors associated with physical activity of patients with osteoarthritis of the lower limb J Eval Clin Pract 2008 14 288 293 10.1111/j.1365-2753.2007.00852.x 18324933

[B24] British Heart Foundation Statistics Database Diet, Physical Activity and Obesity Statistics 2006 http://www.bhf.org.uk

[B25] Stavropoulos-Kalinoglou A Metsios GS Koutedakis Y Nevill AM Douglas KM Jamurtas A van Zanten JJ Labib M Kitas GD Redefining overweight and obesity in rheumatoid arthritis patients Ann Rheum Dis 2007 66 1316 1321 10.1136/ard.2006.060319 17289757 PMC1994320

[B26] Panoulas VF Metsios GS Pace AV John H Treharne GJ Banks MJ Kitas GD Hypertension in rheumatoid arthritis Rheumatology (Oxford) 2008 47 1286 1298 10.1093/rheumatology/ken159 18467370

[B27] Panoulas VF Douglas KM Milionis HJ Stavropoulos-Kalinglou A Nightingale P Kita MD Tselios AL Metsios GS Elisaf MS Kitas GD Prevalence and associations of hypertension and its control in patients with rheumatoid arthritis Rheumatology (Oxford) 2007 46 1477 1482 10.1093/rheumatology/kem169 17704521

[B28] Toms TE Panoulas VF Douglas KM Griffiths H Sattar N Smith JP Symmons DP Nightingale P Metsios GS Kitas GD Statin use in rheumatoid arthritis in relation to actual cardiovascular risk: evidence for substantial under treatment of lipid associated cardiovascular risk? Ann Rheum Dis 2010 69 683 688 10.1136/ard.2009.115717 19854705

[B29] Metsios GS Stavropoulos-Kalinoglou A Panoulas VF Koutedakis Y Nevill AM Douglas KM Kita M Kitas GD New resting energy expenditure prediction equations for patients with rheumatoid arthritis Rheumatology (Oxford) 2008 47 500 506 18304942 10.1093/rheumatology/ken022

[B30] Metsios GS Stavropoulos-Kalinglou A Panoulas VF Koutedakis Y Kitas GD Metabolism in patients with rheumatoid arthritis: resting energy expenditure, physical activity and diet-induced thermogenesis. Invited review Recent Patents Endocrine, Metabolic Immune Drug Discovery 2008 2 97 102 10.2174/187221408784534277

[B31] Brameld KJ Holman CD Demographic factors as predictors for hospital admission in patients with chronic disease Aust N Z J Public Health 2006 30 562 566 10.1111/j.1467-842X.2006.tb00787.x 17209274

